# Supplementary material for: The impact of the COVID-19 pandemic on blood culture practices and bloodstream infections
Source: Microbiol Spectr. 2023 Nov 17;11(6):e02630-23. doi: 10.1128/spectrum.02630-23 (PMC10783801; doi:10.1128/spectrum.02630-23)
Supplement: Supplemental material — Tables S1 to S4; Fig. S1 to S4. [file spectrum.02630-23-s0001.docx]

**Supplemental Figures and Tables**

Supplemental Table S1: Incidence rate ratios of blood culture draws, using segmented regression models, comparing peri-pandemic to pre-pandemic periods.

| **Outcome** | **Incidence Rate Ratio** | **95% Confidence Interval** | **p-value** |
| --- | --- | --- | --- |
| Community Culture Rate | 1.01 | 0.94 – 1.09 | 0.73 |
| Hospital Culture Rate | 1.09 | 1.01 – 1.19 | <0.05 |
| LTC Culture Rate | 0.97 | 0.87 – 1.08 | 0.58 |

Supplemental Table S2: Odds ratios of proportion of CoNS contaminants, using segmented regression models, comparing peri-pandemic to pre-pandemic periods.

| **Setting** | **Odds Ratios** | **95% Confidence Interval** | **p-value** |
| --- | --- | --- | --- |
| Community | 1.07 | 1.02 – 1.13 | <0.01 |
| Hospital | 0.90 | 0.84 – 0.97 | <0.01 |
| LTC | 1.14 | 1.01 – 1.29 | <0.05 |

Supplemental Table S3: Incidence rate ratios of hospital BSI with resistant organisms, using segmented regression models, comparing peri-pandemic to pre-pandemic periods.

| **BSI pathogen** | **Incidence Rate Ratio** | **95% Confidence Interval** | **p-value** |
| --- | --- | --- | --- |
| MRSA | 0.75 | 0.47 – 1.19 | 0.22 |
| VRE | 1.22 | 0.80 – 1.89 | 0.35 |
| ESBL | 0.83 | 0.59 – 1.17 | 0.28 |

Supplemental Table S4: Selected segmented regression model coefficients, comparing peri-pandemic to pre-pandemic periods, with the inclusion of both step and slope change.

| **Outcome** | **Coefficient, overall slope (week)** | | **Coefficient, level change at pandemic onset (pandemic)** | | **Coefficient, slope change at pandemic onset (week*pandemic)** | |
| --- | --- | --- | --- | --- | --- | --- |
|  | Coefficient | 95% CI^a^ | Coefficient | 95% CI | Coefficient | 95% CI |
| Community Culture Ordering Rate | 7.60 x 10^-4^* | 1.30 x 10^-4^  - 1.39 x 10^-3^ | -0.70 | -1.40 – 0.01 | 4.06 x 10 ^-3^* | 7.05 x 10^-5^  - 8.05 x 10^-3^ |
| LTC Culture Ordering Rate | 6.61 x 10^-5^ | -5.23 x 10^-4^ – 6.56 x 10^-4^ | -2.15** | -3.03 – -1.27 | 1.14 x 10^-2^** | 6.64 x 10 ^-3^ – 1.61 x 10 ^-2^ |
| LTC BSI Rate | 6.52 x 10^-4^* | 4.41 x 10 ^-5^ – 1.26 x 10 ^-3^ | -2.55** | -3.52 – -1.58 | 1.28 x 10^-2^** | 7.66 x 10 ^-3^ – 1.80 x 10^-2^ |

^a^ 95% Confidence Interval

* p-value < 0.05

** p-value < 0.01

Supplemental Figure S1: Weekly person-days in hospital (A) and LTC (B) settings

(A)

**
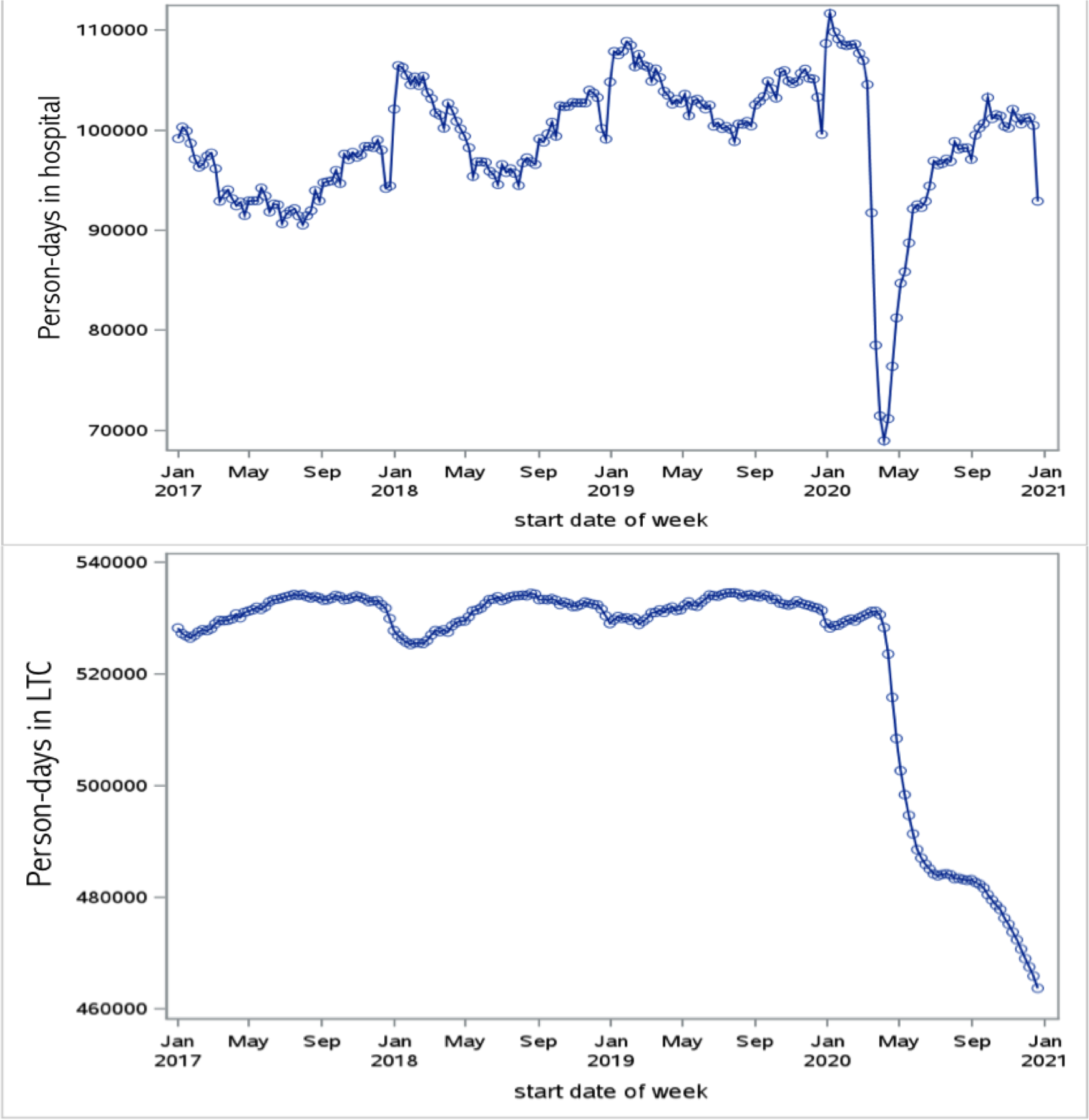
**

(B)

**
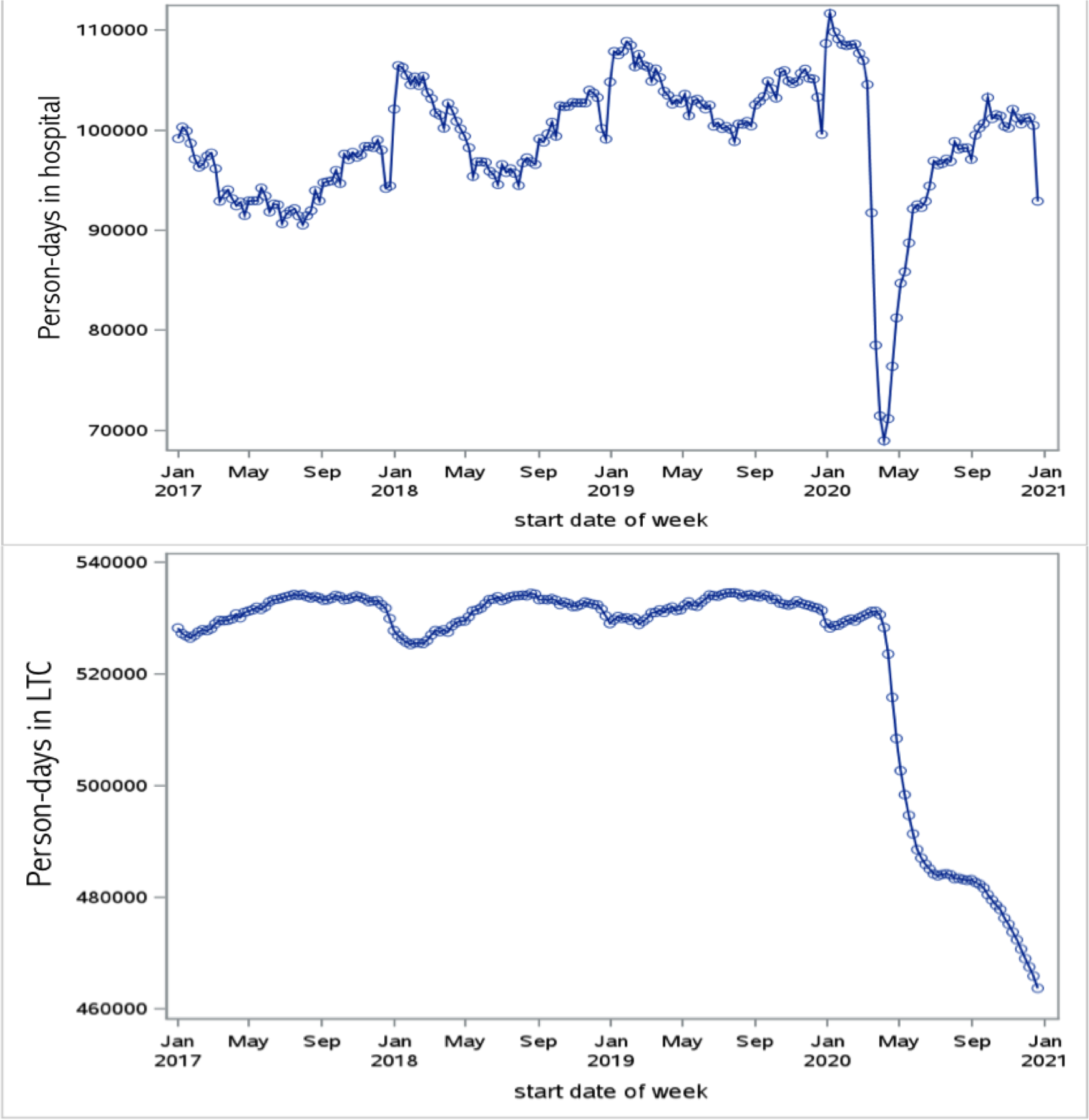
**

Supplemental Figure S2: Incidence of community BSI by organism, by month (A) or week (B)

(A)


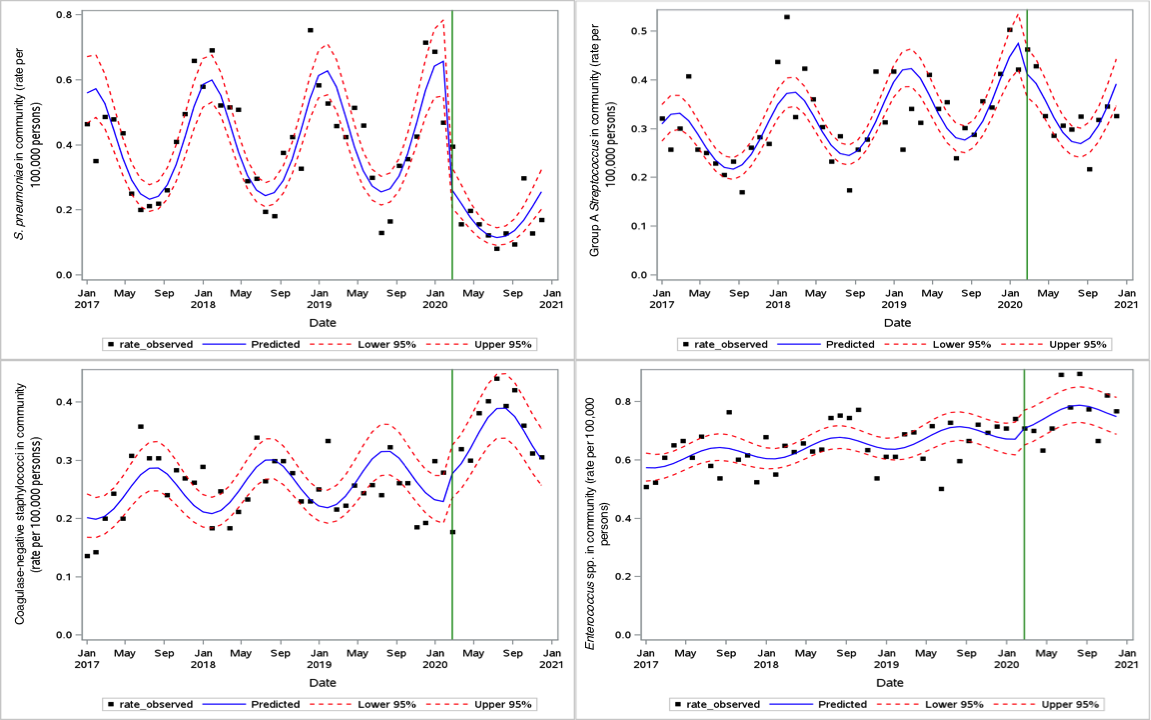


(B)


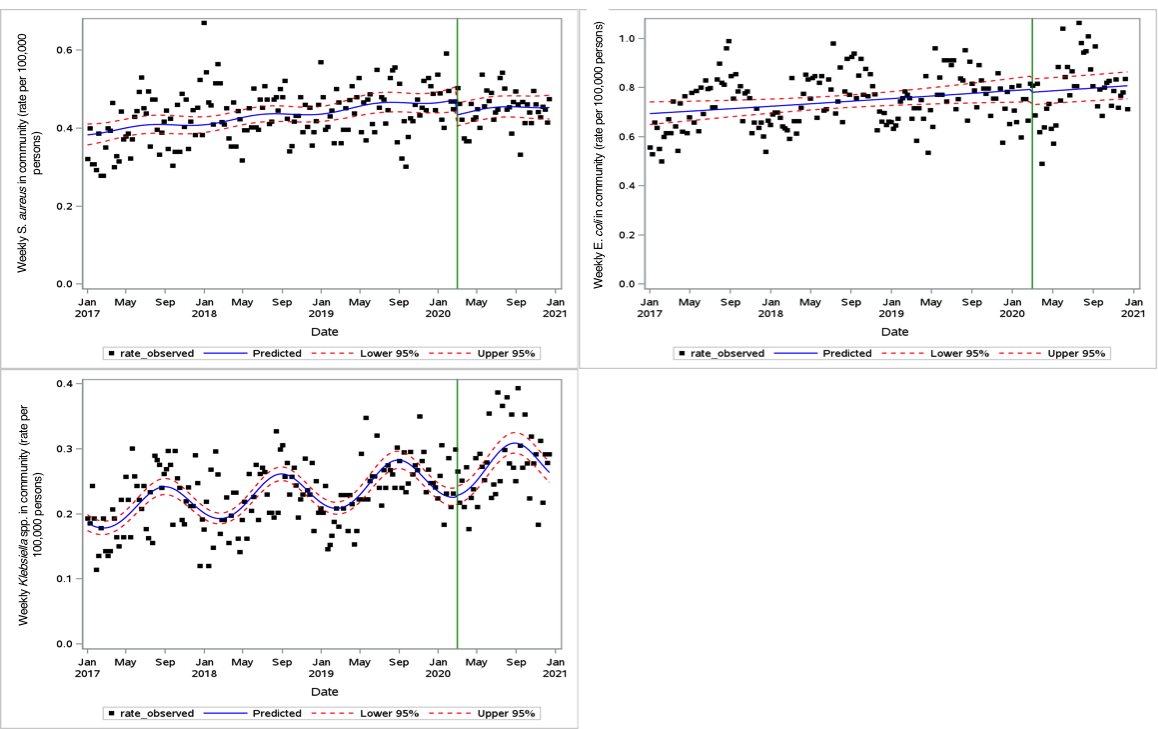


Green vertical line indicates onset of pandemic period (March 2020).

Community *E. coli* BSI modelling did not converge with sine/cosine functions for seasonality.

Supplemental Figure S3: Weekly incidence of hospital BSI by organism.


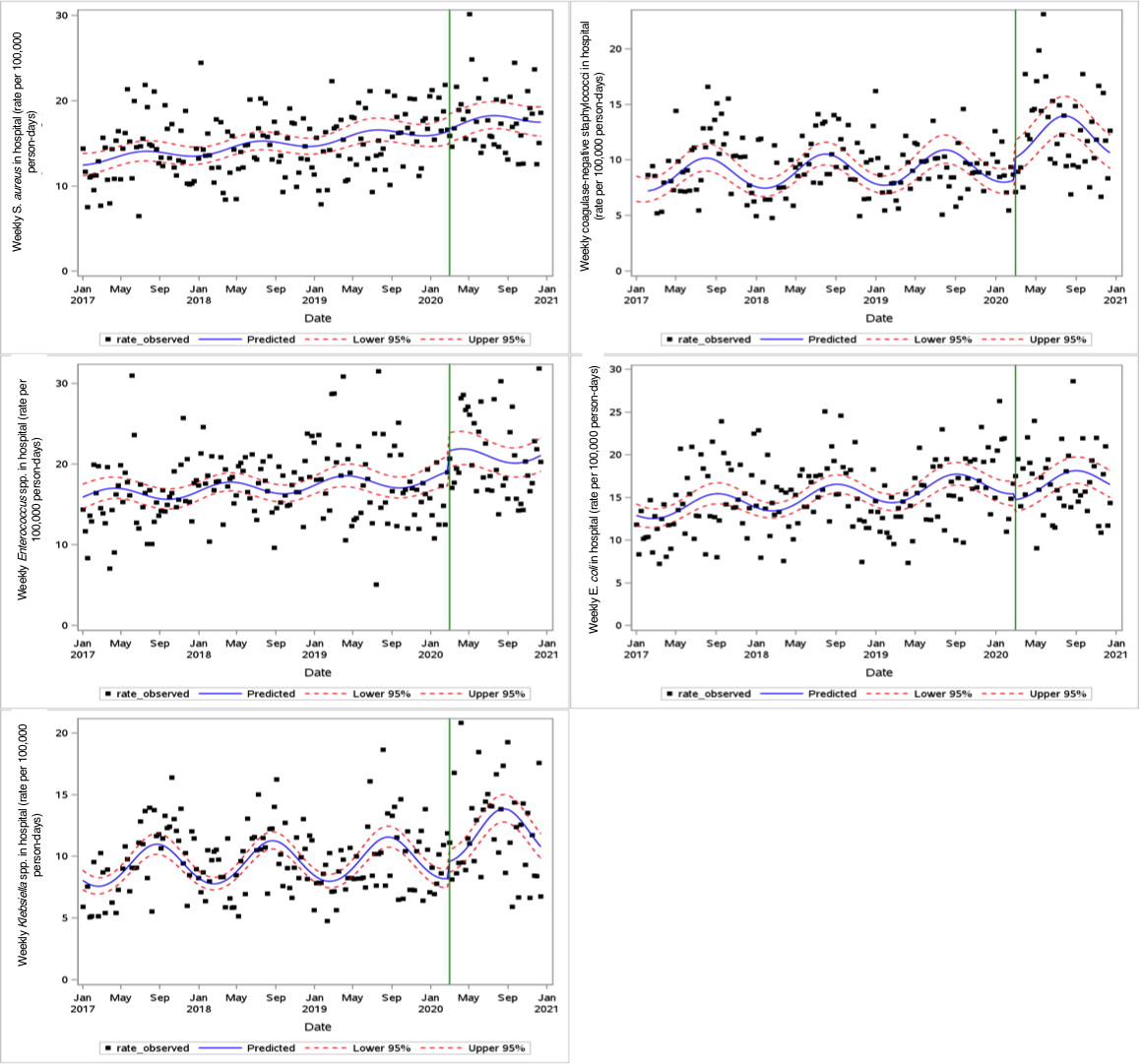


Green vertical line indicates onset of pandemic period (March 2020).

16 time-points censored for low counts (weekly CNS BSI plot)

7 time-points censored for low counts (weekly *Klebsiella* spp. BSI plot)

Supplemental Figure S4: Monthly incidence of LTC-BSI by organism.


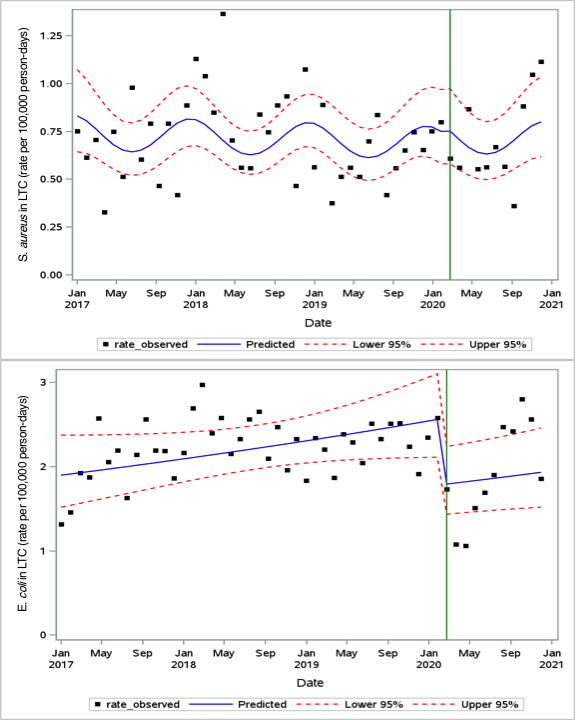


Green vertical line indicates onset of pandemic period (March 2020).
